# Disposable E-Tongue for the Assessment of Water Quality in Fish Tanks

**DOI:** 10.3390/s8063665

**Published:** 2008-06-01

**Authors:** Chew-Cheen Chang, Bahruddin Saad, Misni Surif, Mohd Noor Ahmad, Ali Yeon Md Shakaff

**Affiliations:** 1 School of Chemical Sciences, Universiti Sains Malaysia, 11800 Penang, Malaysia; 2 School of Distance Education, Universiti Sains Malaysia, 11800 Penang, Malaysia; 3 School of Bioprocess Engineering, Universiti Malaysia Perlis, 02600 Perlis, Malaysia; 4 School of Computer and Communication Engineering, Universiti Malaysia Perlis, 02600 Perlis, Malaysia

**Keywords:** E-tongue, lipid, electroactive material, water quality, pattern recognition

## Abstract

A disposable screen-printed e-tongue based on sensor array and pattern recognition that is suitable for the assessment of water quality in fish tanks is described. The characteristics of sensors fabricated using two kinds of sensing materials, namely (i) lipids (referred to as Type 1), and (ii) alternative electroactive materials comprising liquid ion-exchangers and macrocyclic compounds (Type 2) were evaluated for their performance stability, sensitivity and reproducibility. The Type 2 e-tongue was found to have better sensing performance in terms of sensitivity and reproducibility and was thus used for application studies. By using a pattern recognition tool i.e. principal component analysis (PCA), the e-tongue was able to discriminate the changes in the water quality in tilapia and catfish tanks monitored over eight days. E-tongues coupled with partial least squares (PLS) was used for the quantitative analysis of nitrate and ammonium ions in catfish tank water and good agreement were found with the ion-chromatography method (relative error, ±1.04- 4.10 %).

## 1. Introduction

The rearing of fish, cockles, prawns, etc. as alternative source of protein to feed the ever increasing world population is becoming more and more important. In these activities, the monitoring of water quality is essential for a successful operation. Important water quality parameters that affect directly the growth and health of the fish include temperature, pH, dissolved oxygen, carbon dioxide, ammonia, nitrite, nitrate, phosphorus, etc. [1].

Various methods are currently used to monitor these parameters, ranging from simple devices to expensive instruments. Chemical methods such as spectrophotometry and liquid chromatography are widely used, but the analysis is often time-consuming, require sample pretreatment and require skilled operators. Therefore, alternative approaches such as disposable sensor arrays are gaining more attention. This approach capitalizes on the advantageous features of potentiometric sensors, i.e., rapid response, low cost, possibility of on-site analysis using a relatively simple measuring set-up and overcome one of the main drawbacks of conventional sensors that possess inadequate selectivity in multicomponent environments [2, 3].

A sensor array based on lipids was introduced by Toko in 1990 [4]. Also known as the taste sensor or e-tongue, it has found many applications such as in the taste quantification and foodstuffs classification [5]. It has been proposed for the classification of beers, coffee, tea and water [4, 6-8]. An array of non-specific potentiometric chemical sensors based on chalcogenide glass has been reported and tested on foodstuffs, clinical, industrial and environmental samples. [5]. The e-tongue has been applied for the qualitative and quantitative analysis of mineral water and wine [9], recognition of coffee [10], discrimination of fruit juices and monitoring of juice spoilage [11]. A multisensor system coupled with artificial neural networks has been used for the determination of inorganic pollutants in a model groundwater system [2].

In the fabrication of the e-tongue, signal from the low selectivity sensor array are processed using partial least squares (PLS), principal component analysis (PCA), and artificial neural network (ANN) [12] to extract both qualitative and quantitative information. Sensors based on various principles can be employed, the more commonly used being potentiometric, voltammetric, and amperometric types.

Sensing materials used in e-tongues based on potentiometric principles may also vary significantly [12]. Different sensing materials and various sensor arrays together with a variety of analytical strategies have been applied by different authors. Nevertheless, the choice of sensing method depends on the composition of the sample to be examined [13]. Many interesting molecular receptors, crown ethers in particular, have been synthesized as sensing materials. In this work, the suitability of some of these compounds and the liquid ion-exchangers are evaluated as e-tongue when fabricated using the screen-printing technology. The sensing performance of these alternative electroactive materials will be compared to the classical lipid materials originally proposed by Toko *et al.* Such low-cost disposable e-tongue could be useful for water quality monitoring in the aquaculture industry.

## 2. Experimental Section

### 2.1 Reagents and solutions

Chemicals used were purchased from the following sources: high molecular weight poly(vinyl chloride, PVC), oleyl amine (Oam, 76 %), decyl alcohol (DA, >99.5 %), 2-nitrophenyloctyl ether (2-NPOE, 99 %), tridodecylamine (TDDA, hydrogen ionophore I), dibenzo-24-crown-8 (98 %), potassium tetrakis(4-chlorophenyl) borate (KTClPB, 98 %) were from Fluka (Switzerland); tris-ethylhexyl phosphate (TEHP, 97 %), dioctyl phenylphosphonate (DOPP), Aliquat 336 were from Sigma Aldrich (Germany); oleic acid, ammonium sulphate (99.5 %), sodium nitrite (99.5 %), di-sodium hydrogen phosphate (99 %), sodium carbonate (99.9 %), sodium hydrogen carbonate (99.7 % ∼ 100.3 %) and sulfuric acid (95.97 %), 1000 ppm standard solutions of nitrate, nitrite and ammonium ions, tartaric acid (99.5 %), dipicolinic acid were from Merck (Germany); trioctyl methylammonium chloride (TOMA) and dioctyl phosphate (DOP) were from Tokyo Chemicals, Japan; tetrahydrofuran (THF) was from Fisher, UK; dibenzo-18-crown-6 (98 %) was from Acrōs Organics (USA); potassium nitrate (99.5 %) was from Riedel-de Haēn AG (Germany); potassium dihydrogenphosphate was from Univar (Australia). 0.45 μm pore diameter membrane syringe filters were from Whatman (England;. Ultra Pure Water (UPW, 18.2 M Ω/ cm) was used to prepare all solutions.

### 2.2 Disposable e-tongue

**Figure 1.**
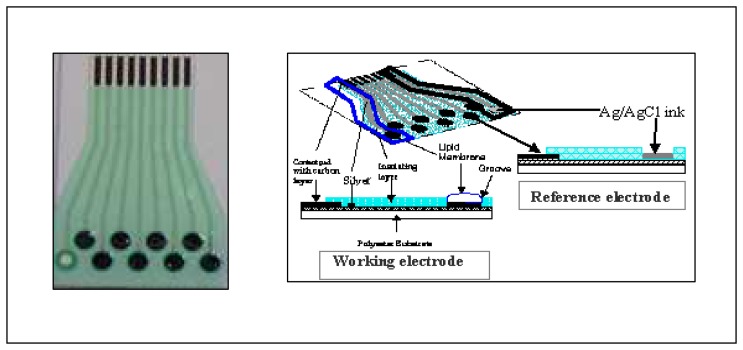
Front and cross-sectional view of disposable sensor strip [14]. a) Front view of sensor strip b) Cross sectional view of sensor strip

The e-tongue consists of eight track working electrodes and one track of reference electrode. It was fabricated by using screen-printing technology and in accordance with a previously reported method [14]. The process was carried out in four consecutive printing steps: (i) nine conducting paths were printed with silver ink (Electrodag® 425A); (ii) nine conducting pads and circular working electrode areas (4 mm diameter) were printed with graphite-based ink (Electrodag® 440); (iii) followed by Ag/AgCl as the reference electrode (4 mm diameter) (Electrodag® 7019); (iv) four insulation layers were then printed on the polyester substrate to create the circular grooves. The final dimension of the layout of the screen-printed strip is 3.8 cm × 5.7 cm. Figure 1 shows the front view and cross-sectional view of the disposable screen-printed e-tongue.

### 2.3 Preparation of disposable e-tongue

Lipid sensing materials as proposed by Toko *et al.* [4] were used to prepare the Type 1 e-tongue. The sensing cocktail consists of lipid materials (50 mg), PVC (170 mg), and DOPP (360 mg) as plasticizer (Table 1). THF (3.0 mL) was used to dissolve the sensing materials and the mixture was stirred for 10 minutes. The sensing cocktails were deposited on the working electrodes by using a high precision fluid dispenser model Σx-V2 from Musashi Engineering. The sensor strip can be used after the slow evaporation (one day) of THF at room temperature. The procedure to prepare Type 2 e-tongue was the same for the Type 1 except that the cocktail compositions were different and THF (1.5 mL) was used to dissolve the sensing materials (Table 1).

### 2.4 Preparation of standard solutions

Standard solutions of KNO_3_, NaNO_2_ and (NH_4_)_2_SO_4_ (10^-8^ M – 10^-1^ M) were serially diluted from 1 M stock solutions. Phosphate buffer solutions with different pH (pH 6.00 - 9.10) were prepared by using appropriate amounts of Na_2_HPO_4_ and KH_2_PO_4_ [15].

### 2.5 Characterization of disposable e-tongue

Potentiometric measurements were performed using an eight-channel high impedance multi-interface meter from Fylde Scientific, U.K. The multi-interface meter (version 2.0 software) was connected to a personal computer and multi-interface for data collection. The potential values were measured versus Ag/AgCl reference electrode for Type 1 and 2 e-tongues.

Stability test was carried out by immersing the sensor strip in 100 mM of NaNO_2_ solutions for 40 minutes and the data recorded every 20 seconds. Prior to measurements, the sensor strip was preconditioned in the standard solution for 1 minute.

The sensitivity of the e-tongues were studied by measuring the responses when the concentrations of the standard solutions (KNO_3_, NaNO_2_ and (NH_4_)_2_SO_4_) was changed from low concentration (10^-8^ M) to high concentration (10^-1^ M); the pH of the phosphate buffer solution was studied over pH 6.00 to pH 9.10. One minute of conditioning was allowed before the data were collected and all measurements were taken for one minute with ten seconds interval for each concentration. The sensor strip was rinsed with water in between measurements.

The discriminative ability of the appropriate e-tongue was studied by measuring 100 mM standard solutions (KNO_3_, NaNO_2_, and (NH_4_)_2_SO_4_) and pH 7.0 phosphate buffer solutions. The measurements were done for one minute and data were recorded every ten seconds. The signals obtained were analyzed by using PCA.

### 2.6 Analysis of water samples

Tilapia *(Oreochromis sp.)* and catfish (*Clarias gariapinus*) were reared in fish tanks (7.5 m height × 9.2 m diameter, 500 m^3^) at the fish house of the School of Biological Sciences, USM. The fish were fed under the recommended feeding schedules over eight days, after that the water was drained and replaced with fresh water. Samples were collected using polyethylene bottles and were analyzed as soon as possible. If not, they were stored in a refrigerator (4 °C) for not more than a week.

Qualitative analysis was carried out by measuring the water samples which were sampled over eight days. Samples were filtered before the measurements, and were measured for its pH value. Nitrite, nitrate and ammonium ions were determined by using ion chromatography.

Quantitative analysis on nitrate and ammonium ions in catfish tank water samples were studied by using the standard addition method. First, the concentration of nitrate was determined by ion chromatography. Standard analyte (1000 ppm, 40 μL) was spiked into the solution thirteen times and the potential readings taken after each addition of analyte. The same procedure was repeated for the determination of ammonium ion, except 10 μL of standard analyte (1000 ppm) was spiked into the solution nine times. Tables 2 and 3 show the training model solutions for nitrate and ammonium ions, respectively. Calibration model for each analyte was built by using partial least square (PLS). Multivariate calibration was performed twice for each analyte. Five test samples were measured (n=2) and the obtained data was used for the prediction based on the established calibration model. The results predicted by the e-tongue were compared with ion chromatography.

### 2.7 Data processing

The classification and monitoring of water quality on different days was performed using multivariate data analysis, principal component analysis (PCA). Partial least squares (PLS) from The Unscrambler (v 8.0.5, Camo, Norway) was used to analyze the quantitative data. Data were standardized prior to the modeling. Calibration model for nitrate and ammonium ions was built separately. Full cross validation was applied to test the calibration model [16]. The predicted values were based on the calibration model.

### 2.8 Ion chromatography

A Metrohm model 792 IC with chemical suppression for anions using a Metrohm Suppressor Module (MSM) was used. The chromatographic conditions used are shown in Table 4. Sulfuric acid was used as the regenerant for the anion conductivity suppressor device. Mixed anion standard solutions (NO_2_^-^, NO_3_^-^) (0.1 ppm – 20 ppm) were prepared by dilution of the stock solutions (1000 ppm). (NH_4_^+^) (0.5 – 5 ppm) were prepared from stock solutions (1000 ppm). Membrane syringe filters with 0.45 μm pore diameter were used to filter solutions.

## 3. Results and Discussion

Screen-printing disposable sensors are recommended [17] to overcome the ‘electrode fouling’ phenomenon which is one of the main drawbacks of normal chemical sensors [18]. Both the Type 1 and 2 e-tongues were found to produce stable potential readings (% RSD, 0.46-5.36 % and 0.29-1.21 %, respectively) over the tested duration.

Apart from stability, it is also important for the e-tongue to have the required sensitivity to discriminate between the different samples [18]. In a multicomponent environment, the selectivity of most sensors is not sufficiently selective and normally the ‘selectivity’ response is hardly considered [9]. However, valuable information can still be obtained from the non-selective sensor [9].

The slopes of the calibration curves when measured in NaNO_2_, KNO_3_ and (NH_4_)_2_SO_4_ and phosphate buffer solutions are shown in Table 5.

It can be readily seen that the Type 2 e-tongue has overall better sensitivities then the Type 1, especially in NaNO_2_ solutions where near-Nernstian slopes were obtained. Channels 1, 2 and 6 of the Type 2 e-tongue containing crown ethers produce near-Nernstian responses towards sodium ions, while channels 3 and 5 that contained the anion exchanger show significant response towards nitrate ions. Crown ethers have been demonstrated to be highly selective complexing agents for many ions such as sodium and potassium [20]. The potential of membrane based on Aliquat 336 (Channels 3 and 5) shows significant response to the anions. Aliquat 336 has also been used for the detection of nitrite and nitrate ions in previous works [21-22]. Membrane that contained tridodecylamine (Channel 7) is a proton-selective ionophore [23] and shows significant response to phosphate buffer solution. The membrane containing the plasticizer DOPP (Channel 4) is known to show selectivity towards cations [24]. KTClPB has the ability to induce anionic sites in the membrane [25].

The reproducibility of the slopes as reflected by the relative standard deviations indicates that the Type 2 e-tongue (RSD range 0.59 – 4.73 %) is more superior to the Type 1 e-tongue (RSD range, 1.00 – 12.97 %). Due to the favourable properties such as higher sensitivity and reproducibility, Type 2 e-tongue was used for the remaining studies.

**Figure 2.**
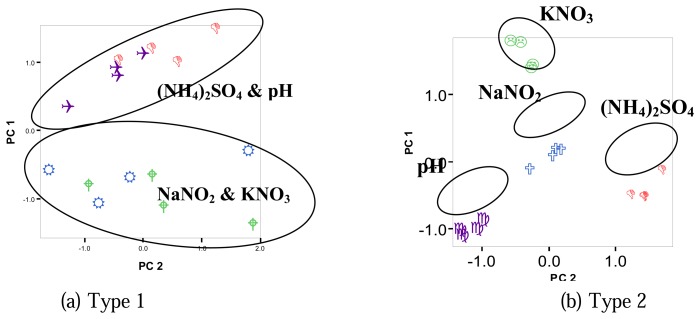
Discrimination of standard solutions by using Type 1 and 2 e-tongues.

The capabilities of Type 1 and 2 e-tongues to discriminate standard solutions were performed and results are illustrated in Figure 2. The PCA plots show that the Type 2 sensor effectively discriminates 100 mM of KNO_3_, NaNO_2_, (NH_4_)_2_SO_4_ and pH 7.0 buffer solutions. The first three principal components PC1 (52.1 %), PC2 (25.5 %), PC3 (15.4 %) explain 93 % of total system variance and all the standard solutions are clearly differentiated in the plots.

The Type 2 e-tongue, coupled with PCA was used for the monitoring of water quality for tilapia and catfish tank samples over eight days.

**Figure 3.**
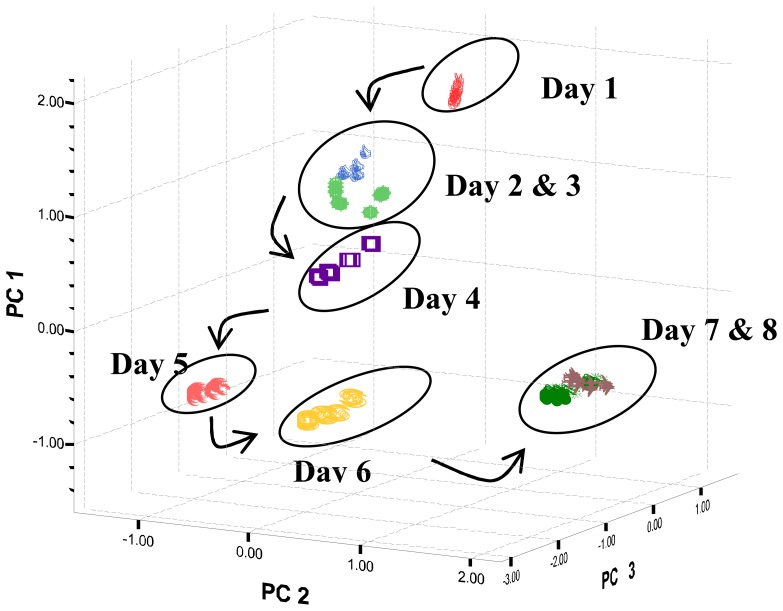
Water quality changes of tilapia water

**Figure 4.**
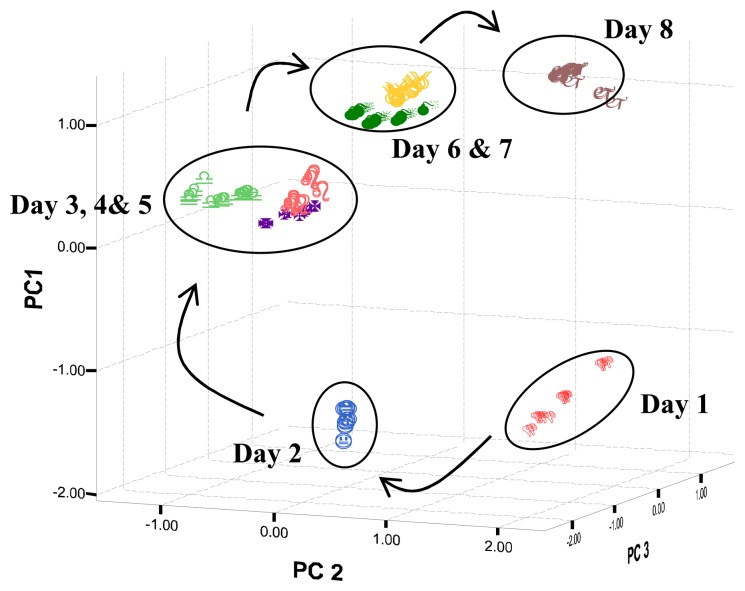
Water quality changes of catfish water

From the PCA plots, it can be found that water samples can be differentiated by the e-tongue (Figures 3 and 4). Table 6 shows the concentration of nitrite, nitrate and ammonium ions as determined by ion chromatography and pH of the water samples over this period.

The concentration of nitrite, nitrate and ammonium ions increases from day 1 to day 8 (Table 6), indicating the gradual changes of the water quality. The water quality deteriorated due to the accumulation of feed residues and from the excretion of fish [26].

For quantitative analysis, the potential of the e-tongue to predict the concentration of nitrate and ammonium ions in the catfish tank water was investigated and compared to ion chromatography. A calibration of the sensor array is required in order to predict the concentration of analyte in the sample [27]. The calibration models were made using multivariate calibration i.e. PLS. Model solutions obtained by using the standard addition method was used for the calibration of the e-tongue. The standard addition method can overcome some of the matrix effects [28]. Calibration models were validated by full cross validation method, leaving out one sample at a time. The number of components is optimal when the number has the lowest prediction variance. The root mean square error of prediction (RMSEP) is the average prediction error of the test sample, and is estimated in the validation stage. Separate calibration model was made for each analyte. A good model will have a slope that is close to 1, a correlation close to 1 and an offset close to 0 [15].

Results from Table 7 show that the calibration model for nitrate and ammonium ion has fulfilled these requirements. The number of components for nitrate and ammonium ions calibration model is three and four, respectively. After obtaining the calibration model, five water samples were used to test the model. The relative error between the concentrations of water samples that was predicted by the e-tongue and determined by ion chromatography are shown in Table 8. Relative error for nitrate ion range from 1.26-4.49 % while for ammonium ion was 1.04-3.45 %.

## 4. Conclusions

Disposable screen-printed e-tongues were fabricated and characterized in terms of stability, sensitivity and reproducibility of signals. The Type 2 e-tongue, based on alternative electroactive materials, showed better sensitivity and reproducibility and was used for subsequent studies. It was not only able to discriminate the standard solutions but also to monitor changes in water quality over eight days. The e-tongue was also able to predict the concentration of nitrate and ammonium ions, with relative errors in the 1.26 – 4.49 % and 1.04 – 3.45 % range, respectively. Due to these favourable features, the Type 2 e-tongue can be recommended for adoption in aquaculture applications.

## Figures and Tables

**Table 1.** Composition of materials used for the fabrication of disposable e-tongues.

**Sensor Channel**	**Type 1**	**Type 2**
**1**	Decyl alcohol (DA) (50.0 mg), DOPP (360.0 mg), PVC (170.0 mg)	Dibenzo-18-crown-6 (5.0 mg), 2-NPOE (61.0 mg), PVC (31.0 mg)
**2**	Oleic acid (OA) (50.0 mg), DOPP (360.0 mg), PVC (170.0 mg)	Dibenzo-24-crown-8 (5.0 mg), 2-NPOE (61.0 mg), PVC (31.0 mg)
**3**	Dioctyl phosphate (DOP) (50.0 mg), DOPP (360.0 mg), PVC (170.0 mg)	Aliquat 336 (5.0 mg), TEHP (61.0 mg), PVC (31.0 mg)
**4**	DOP:TOMA = 9:1 (45.0:5.0 mg), DOPP (360.0 mg), PVC (170.0 mg)	DOPP (66.0 mg), PVC (31.0 mg)
**5**	DOP:TOMA = 5:5 (25.0:25.0 mg), DOPP (360.0 mg), PVC (170.0 mg)	Aliquat 336 (5.0 mg), 2-NPOE (61.0 mg), PVC (31.0 mg)
**6**	DOP:TOMA = 3:7 (15.0:35.0 mg), DOPP (360.0 mg), PVC (170.0 mg)	Dibenzo-18-crown-6 (5.0 mg), TEHP (61.0 mg), PVC (31.0 mg)
**7**	Trioctylmethylammonium chloride (TOMA) (50.0 mg), DOPP (360.0 mg), PVC (170.0 mg)	Tridodecylamine (5.0 mg), 2-NPOE (61.0 mg), PVC (31.0 mg)
**8**	Oleylamine (Oam) (50.0 mg), DOPP (360.0 mg), PVC (170.0 mg)	KTClPB (5.0 mg), 2-NPOE (61.0 mg), PVC (31.0 mg)

**Table 2.** Training model solutions used for multivariate calibration of the e-tongue for the determination of nitrate ion.

**Number of measurement**	Volume of standard spiked (μL)*	**Total volume (mL)**	**Concentration of standard after spiking (ppm)**

**Initial solution : Catfish tank water**			
1	0	25.00	2.40
2	40	25.04	4.00
3	40	25.08	5.58
4	40	25.12	7.16
5	40	25.16	8.74
6	40	25.20	10.32
7	40	25.24	11.88
8	40	25.28	13.45
9	40	25.32	15.01
10	40	25.36	16.56
11	40	25.40	18.08
12	40	25.44	19.62
13	40	25.48	21.16

* 1000 ppm of standard solution was spiked

**Table 3.** Training model solutions used for multivariate calibration of e-tongue for the determination of ammonium ion.

**Number of measurement**	Volume of standard spiked (μL)*	**Total volume (mL)**	**Concentration of standard after spiking (ppm)**

**Initial solution : Catfish tank water**			
1	0	25.00	0.44
2	10	25.01	0.84
3	10	25.02	1.24
4	10	25.03	1.63
5	10	25.04	2.03
6	10	25.05	2.43
7	10	25.06	2.83
8	10	25.07	3.23
9	10	25.08	3.62

* 1000 ppm of standard solution was spiked

**Table 4.** Ion chromatographic system used

	**Anion system**	**Cation system**
**Separator column**	Metrosep A Supp 5 -150 (4.0 × 150 mm)	Metrosep C 2 -150 (4.0 × 150 mm)
**Guard column (precolumn)**	Metrosep A Supp 4/5 Guard (3.1 × 29 mm)	Metrosep C2 (4 × 5 mm)
**Eluent solution**	NaHCO_3_, 1.0 mM Na_2_CO_3_, 3.2 mM	Tartaric acid, 4.0 mM Dipicolinic acid, 0.75 mM
**Sample loop size (μL)**	20	20
**Eluent flow rate (mL min^-1^)**	0.7	1.0

**Table 5.** Slope of the channels of e-tongues Type 1 and Type 2 when calibrated in different solutions.

**Channel**	**KNO_3_**	**NaNO_2_**	**(NH_4_)_2_SO_4_**	**Buffer solutions**
**1**	36.4±0.9	55.2±0.6	53.1±1.9	-9.2±0.4
	(45.8±1.2)	(50.0±0.7)	(51.1±0.9)	(-1.7±0.1)
**2**	27.2±1.4	44.9±2	49.5±1.7	-10.4±0.3
	(33.7±1.1)	(52.0±1.1)	(54.6±0.8)	(-1.2±0.1)
**3**	5.9±0.7	15.1±0.7	17.3±0.4	-13.4±1.6
	(-51.6±0.8)	(-42.8±0.7)	(-11.6±0.4)	(6.2±0.2)
**4**	4.1±0.4	13.3±1.2	4.0±0.3	-12.9±0.5
	(37.5±1.0)	(44.9±1.4)	(36.2±0.5)	(-4.2±0.1)
**5**	-11.2±0.3	u.a	u.a	-10.0±0.2
	(-60.0±2.6)	(-50.6±0.9)	(-17.5±0.3)	(-5.3±0.2)
**6**	-42.0±0.8	-31.4±1.9	u.a	17.0±1.1
	(49.5±0.7)	(58.6±0.6)	(46.5±0.4)	(3.9±0.2)
**7**	-66.6±1.0	-45.8±1.2	-18.8±0.8	-3.5±0.4
	(-16.7±0.5)	(-28.4±0.7)	(3.7±0.1)	(48.5±0.6)
**8**	-36.5±1.1	-31. 8± 1.8	u.a	39.3±2.0
	(42.7±2.0)	(35.9±0.8)	(46.1±0.3)	(-6.3±0.1)

* Data for Type 2 e-tongue are shown in parentheses; Standard deviation of the slope is shown as ±; u.a – unavailable (cannot be determined).

**Table 6.** Concentration of nitrite, nitrate and ammonium ions for water samples (n=2)

**Water sample**	**NO_2_^-^(ppm)**	**NO_3_^-^(ppm)**	**NH_4_^+^(ppm)**	**pH**
**a) Tilapia Tank**				
				
**Day 1**	0	1.39	0.81	7.60
**Day 4**	0.18	1.62	1.60	7.38
**Day 8**	0.30	2.72	3.87	6.30
**b) Catfish Tank**				
				
**Day 1**	0	1.05	0.66	7.70
**Day 4**	0.25	1.49	1.47	6.93
**Day 8**	0.32	1.78	3.44	6.67

**Table 7.** Results of partial least squares analysis (n=2).

	**Correlation**	**Slope**	**Offset**	**RMSEC/RMSEP**
**a) Nitrate ion**				
				
**Calibration**	0.9998	0.9997	0.0041	0.1094
**Validation**	0.9995	1.0007	-0.1268	0.1968
**b) Ammonium ion**				
				
**Calibration**	0.9987	0.9973	0.0054	0.0531
**Validation**	0.9964	1.0177	-0.0584	0.0935

**Table 8.** Results of the determination of nitrate and ammonium ions in catfish water sample (n=2).

**Water samples**	**Predicted (ppm)**	**Measured (ppm)**	**Relative error (%)**
**a) Nitrate ion**			
				
1	3.98	4.15	-4.10
2	10.20	10.36	-1.54
3	3.26	3.12	4.49
4	8.85	8.74	1.26
5	13.52	13.20	2.42
**b) Ammonium ion**			
				
1	1.17	1.21	-3.31
2	1.40	1.44	-2.78
3	2.86	2.89	-1.04
4	0.80	0.79	1.27
5	0.60	0.58	3.45
